# Urethral Metastasis of Therapy‐Induced Neuroendocrine Prostate Cancer Successfully Managed With Chemoradiotherapy: A Case Report

**DOI:** 10.1002/iju5.70234

**Published:** 2026-08-07

**Authors:** Toshiaki Kayaba, Satoshi Washino, Takanori Hayase, Kai Yazaki, Kimitoshi Saito, Tomoaki Miyagawa

**Affiliations:** ^1^ Department of Urology Jichi Medical University Saitama Medical Center Saitama Saitama Japan

**Keywords:** case report, castration‐resistant prostate cancer, chemoradiotherapy, neuroendocrine prostate cancer, urethral metastasis

## Abstract

**Introduction:**

Urethral metastasis of prostate cancer is exceptionally rare, particularly in castration‐resistant disease. Therapy‐induced neuroendocrine prostate cancer (t‐NEPC) is an aggressive variant with limited treatment options.

**Case Presentation:**

A 72‐year‐old man, 14 years after diagnosis of prostate adenocarcinoma treated with androgen deprivation therapy, presented with hematuria and dysuria. Prostate‐specific antigen was 2.05 ng/mL and neuron‐specific enolase was 28.0 ng/mL. Urethroscopy revealed papillary tumors in the pendulous urethra. Histology showed high‐grade adenocarcinoma with a small cell carcinoma component, positive for synaptophysin and prostate‐specific antigen. Genomic profiling demonstrated PTEN loss, TP53 mutation, and androgen receptor amplification. Platinum‐based chemotherapy with external‐beam radiotherapy achieved durable urethral local control despite later distant progression; the patient survived 2 years 10 months.

**Conclusion:**

Platinum‐based chemoradiotherapy may provide durable local control of urethral metastasis from t‐NEPC.

## Keynote Message

Urethral metastasis of prostate cancer is exceptionally rare and typically associated with poorly differentiated or neuroendocrine variants. We report a case of therapy‐induced neuroendocrine prostate cancer with urethral metastasis treated with platinum‐based chemotherapy and external‐beam radiotherapy. Durable urethral control was achieved despite later distant progression, suggesting a role for multimodal local therapy.

## 1 Introduction

Urethral metastasis of prostate cancer is exceptionally rare and even less common in castration‐resistant prostate cancer (CRPC) [1, 2, 3, 4]. Neuroendocrine prostate cancer (NEPC) is an aggressive variant arising de novo in a small subset of patients or, more commonly, as therapy‐induced NEPC (t‐NEPC) during long‐term androgen‐receptor–targeted therapy [5, 6]. It shows low prostate‐specific antigen (PSA) relative to disease burden, elevated neuroendocrine markers, and resistance to androgen‐receptor–targeted agents, with a median cancer‐specific survival of approximately 7 months [6, 7]. Optimal management is undefined; platinum‐based chemotherapy is most often used, and limited evidence supports radiotherapy [7, 8, 9]. We report urethral metastasis of t‐NEPC managed with platinum‐based chemoradiotherapy, with durable urethral control and prolonged survival.

## 2 Case Presentation

**FIGURE 1:**
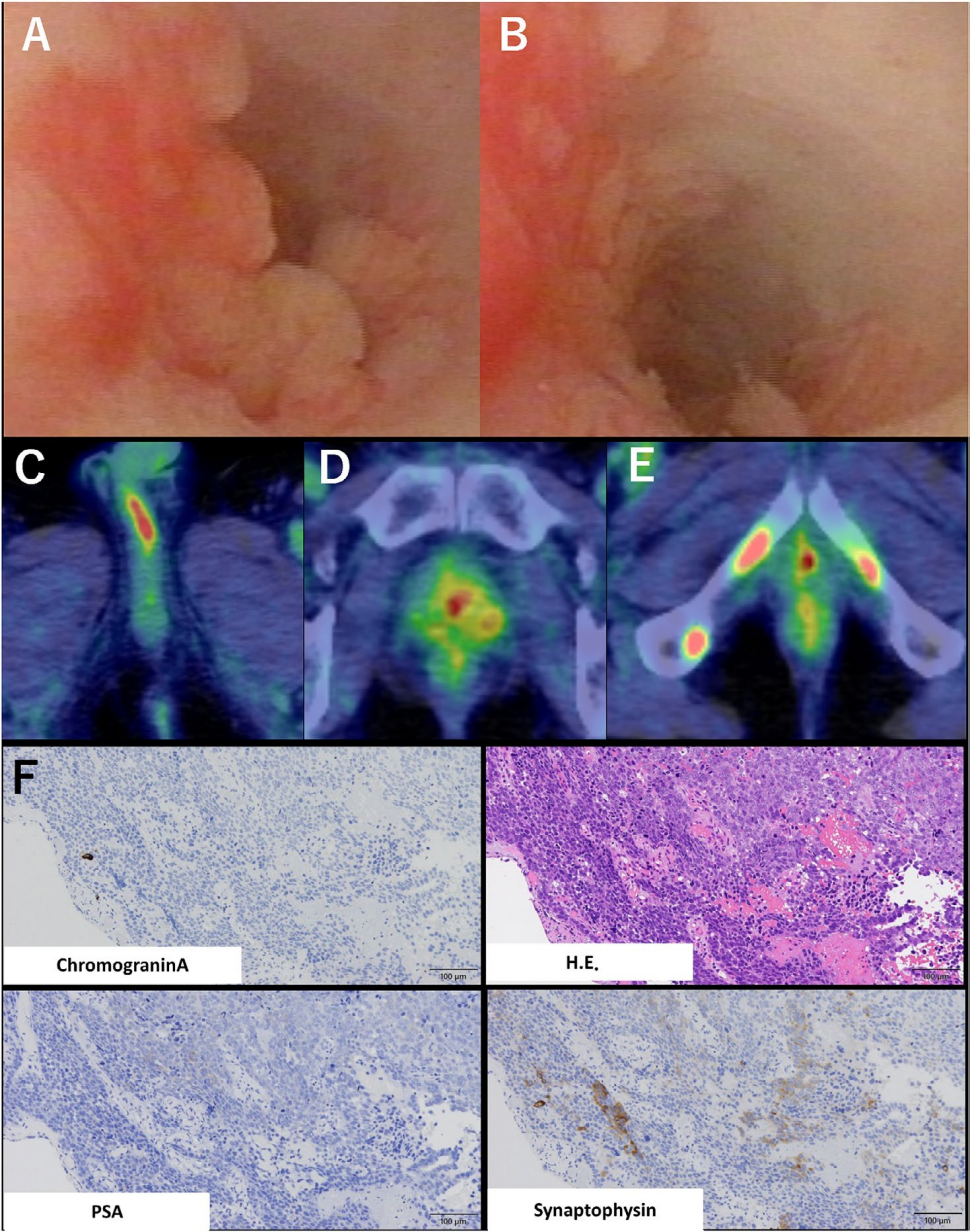
Clinicopathological findings before chemoradiotherapy. (A, B) Urethroscopy showing papillary tumors in the pendulous urethra. (C–E) FDG‐PET‐CT showing abnormal FDG uptake in the urethra (C), prostate (D), and ischium (E). (F) Hematoxylin and eosin staining of the urethral tumor showing high‐grade adenocarcinoma with a minor small cell carcinoma component; immunohistochemistry was positive for prostate‐specific antigen and synaptophysin and negative for chromogranin A. FDG‐PET‐CT, 18F‐fluorodeoxyglucose positron emission tomography–computed tomography.

A 58‐year‐old man was diagnosed with prostate adenocarcinoma (PSA 21.0 ng/mL, Gleason 3 + 3 [1/10 cores], cT1cN0M0, prostate volume 20.7 cm^3^). Because of concurrent pulmonary metastases of renal cell carcinoma being treated with interferon‐alpha and interleukin‐2, he received androgen deprivation therapy (ADT) alone; PSA nadir was 0.166 ng/mL. Biochemical recurrence occurred 8 years after initiation of ADT. However, treatment of the renal cell carcinoma, sorafenib, was prioritized, and ADT was continued. Bicalutamide was added 12 years after treatment initiation for biochemical progression. Thereafter, PSA gradually increased under combined androgen blockade, and 14 years after initiation of ADT he, at 72 years of age, presented with gross hematuria and dysuria. His PSA level was 2.05 ng/mL and neuron‐specific enolase (NSE) 28.0 ng/mL (normal range: ≤ 16.3 ng/mL). Urine cytology demonstrated small cell carcinoma. Urethrocystoscopy identified papillary tumors in the pendulous urethra without visible disease in the prostate or bladder (Figure 1A,B). 18F‐fluorodeoxyglucose positron emission tomography–computed tomography (FDG‐PET‐CT) showed abnormal uptake in the urethra, prostate, and ischium (Figure 1C–E).

Transurethral resection of the urethral tumor and transrectal ultrasound–guided prostate biopsy revealed Gleason 5 + 4 adenocarcinoma (90%) with a small cell carcinoma component (10%) in the urethra and the prostate (Figure 1F). The urethral tumor was positive for synaptophysin and PSA on immunohistochemistry, consistent with neuroendocrine differentiation. Urethral metastasis of CRPC with neuroendocrine differentiation (t‐NEPC) was diagnosed.

**FIGURE 2:**
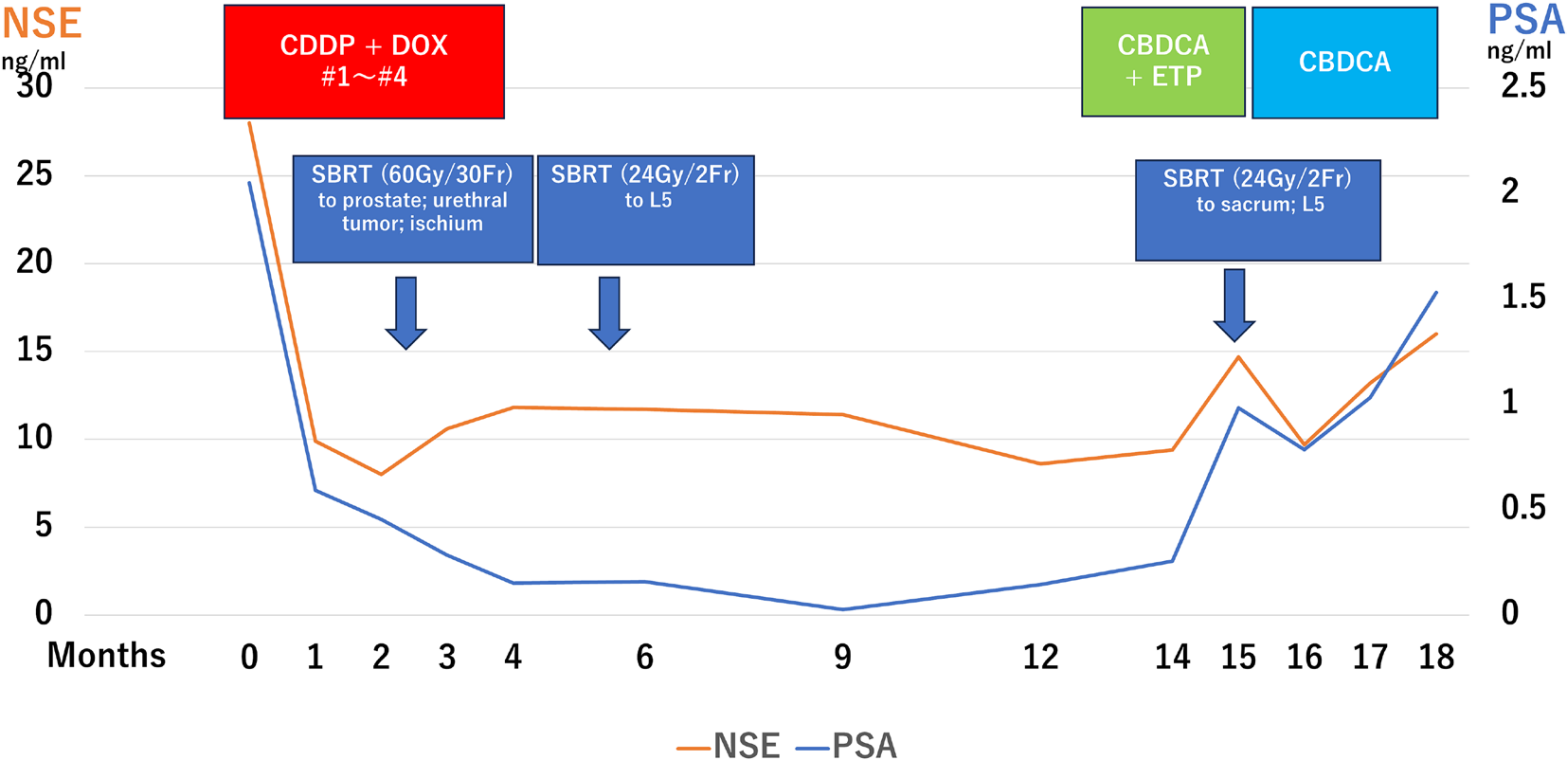
Clinical course showing the sequence of treatments for neuroendocrine prostate cancer (NEPC) and changes in serum prostate‐specific antigen (PSA) and neuron‐specific enolase (NSE). CBDCA, carboplatin; CDDP, cisplatin; DOX, doxorubicin; ETP, etoposide; SBRT, stereotactic body radiotherapy.

**FIGURE 3:**
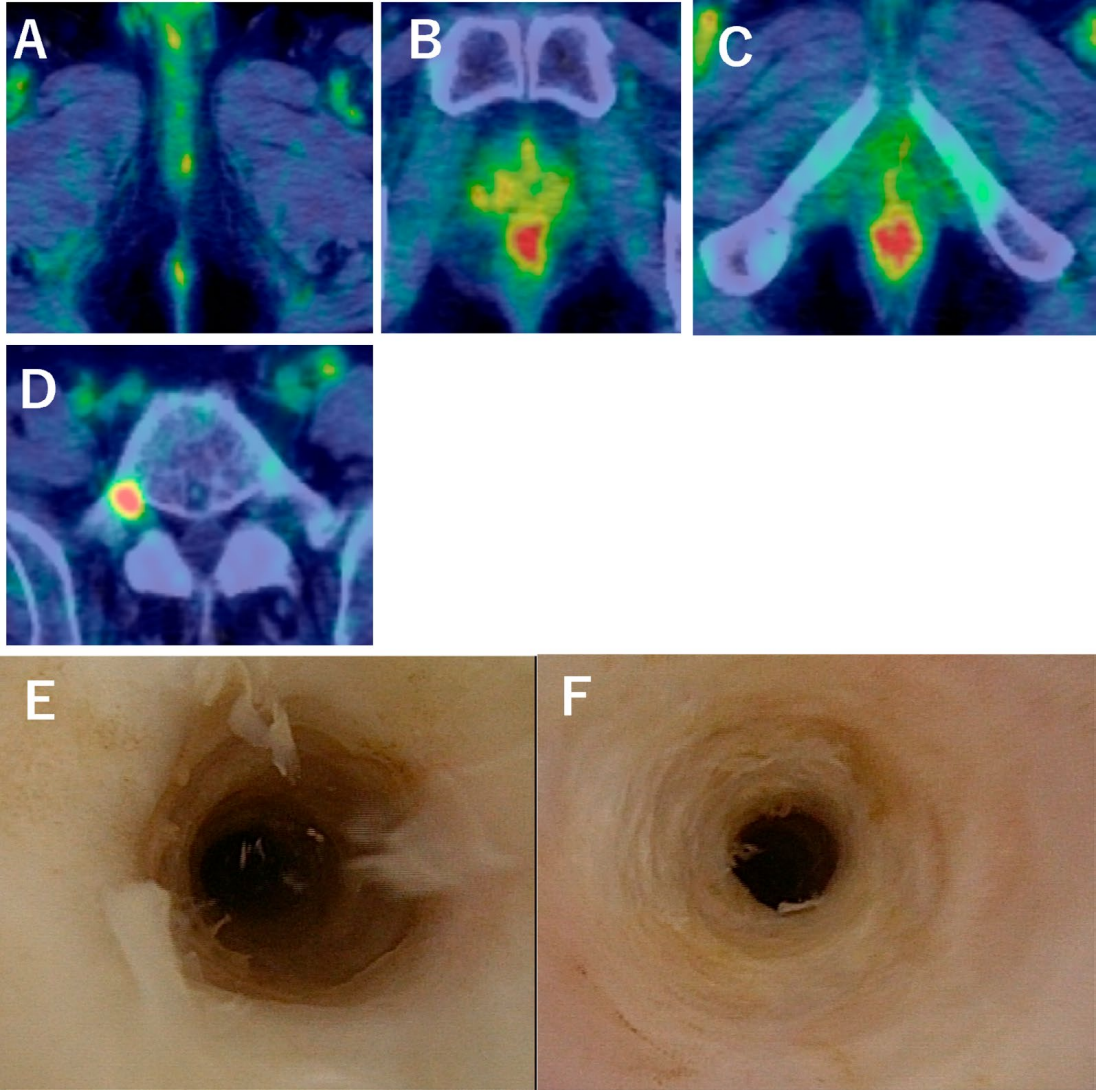
Clinicopathological findings after chemoradiotherapy. (A–C) FDG‐PET‐CT showing reduced FDG uptake in the prostate (A), urethra (B), and ischium (C). (D) New FDG uptake in the fifth lumbar (L5) vertebra. (E, F) Follow‐up urethroscopy showing no residual tumor in the pendulous urethra. FDG‐PET‐CT, 18F‐fluorodeoxyglucose positron emission tomography–computed tomography.

The patient received four cycles of cisplatin (30 mg/m^2^) plus doxorubicin (70 mg/m^2^) over 4 months, with concurrent external‐beam radiotherapy (60 Gy in 30 fractions) to the prostate, urethra, and ischial metastasis (Figure 2). The patient experienced grade 3 dermatitis of his penis and grade 2 acute urethritis (dysuria and frequency) during chemoradiotherapy, leading to a temporary 1‐week interruption of radiotherapy; the symptoms resolved with symptomatic management. Post‐treatment FDG‐PET‐CT showed reduced uptake at all three sites (Figure 3A–C) but new uptake in the fifth lumbar (L5) vertebra (Figure 3D). Because of declining renal function, systemic chemotherapy was withheld and stereotactic body radiotherapy (SBRT, 24 Gy in 2 fractions) was delivered to L5, with subsequent reduction in uptake. PSA and NSE were 0.16 and 11.7 ng/mL, respectively.

Comprehensive genomic profiling (FoundationOne CDx; Foundation Medicine, Cambridge, MA, USA) revealed PTEN loss, TP53 mutation, and androgen receptor amplification. One year later, the L5 lesion progressed. Because renal function had deteriorated following cisplatin treatment (eGFR 63.0 mL/min/1.73 m^2^ before cisplatin vs. 38.7 mL/min/1.73 m^2^ after treatment), we changed the chemotherapy regimen to carboplatin (AUC 3.5) plus etoposide (55 mg/m^2^). However, this regimen was stopped after one cycle because of grade 3 hepatic dysfunction. Imaging then showed progression at L5 and a new sacral lesion, while urethroscopy showed no recurrence in the urethra or prostate (Figure 3E,F). A second attempt at carboplatin–etoposide was again limited by grade 3 hepatic toxicity. SBRT was delivered to the sacrum and L5, followed by four courses of single‐agent carboplatin (Figure 2). Subsequent progression in the lungs and hilar lymph nodes (origin not histologically confirmed) was managed with best supportive care. The patient died 2 years 10 months after initiation of treatment for t‐NEPC and remained free of dysuria or hematuria from completion of urethral chemoradiotherapy until death.

## 3 Discussion

Urethral metastasis of prostate cancer is exceptionally uncommon, with fewer than 25 reported cases, and only eight—including the present one—have been documented in CRPC (Table 1) [1, 2, 3, 4]. Reported features include gross hematuria, difficulty urinating, and penile pain, with tumors usually in the pendulous or bulbar urethra. Histology was typically poorly differentiated adenocarcinoma or showed neuroendocrine differentiation, and PSA at presentation was often low (< 20 ng/mL in five of eight cases). These observations suggest that poor differentiation and CRPC‐to‐NEPC transition may predispose to urethral metastasis. Consistent with this, our case harbored a small cell carcinoma component and PTEN loss with TP53 mutation—features characteristic of aggressive variant prostate cancer [5].

**TABLE 1:** Reported cases of urethral metastasis of castration‐resistant prostate cancer, including the present case (*n* = 8).

Cases	Author	Age	Symptoms	PSA levels, ng/mL	Tumor location	Pathology (genomic profile)	Treatment #1	Treatment #2	Progression of urethral meta	Outcomes/survival
1	Jhaveri H	82	Gross hematuria; difficulty urinating	0.26	Membranous and entire anterior urethra	Poorly differentiated adenocarcinoma; negative for PSA in immunostaining	Best supportive care	NA	NA	NA
2	Masuda T	79	Gross hematuria; difficulty urinating	841	Entire anterior urethra	Adenocarcinoma with aggressive variants (TP53 mutation; AR and KRAS amplification; LOH of BRCA2)	Transurethral resection	Chemotherapy (CDDP + ETP)	NA	PD after two cycles of the treatment #2
3	Gomez E	70	Gross hematuria; difficulty urinating	1.7	Pendulous and bulbar urethra	Adenocarcinoma, Gleason score 5 + 5	Transurethral resection	Anterior urethrectomy + vesicostomy	No	Survived at 6 months after the treatment #2
4	Nabi G	65	Gross hematuria	12	Pendulous and bulbar urethra	Adenocarcinoma	NA	NA	NA	NA
5	Kotecha N	64	Urethral bleeding	10.7	Pendulous urethra	Adenocarcinoma	Transurethral resection	NA	No	NA
6	Sabharwal K	75	Difficulty urinating; perineal pain	NA	Pendulous and bulbar urethra	Neuroendocrine differentiation: negative for PSA; positive for synaptophysin and neuron‐specific enolase in immunostaining	Chemotherapy (CBDCA + ETP)	Chemotherapy (CPT‐11 + ETP) + radiotherapy	NA	Died at 10 months
7	Jindal T	66	Penile pain	27.3	Entire anterior urethra	Adenocarcinoma	Chemotherapy (DTX)	NA	No	Survived after 10 cycles of DTX
8	Present case	72	Gross hematuria	2.05	Pendulous urethra	Adenocarcinoma > small cell carcinoma; positive for synaptophysin and PSA in immunostaining (TP53 mutation; PTEN loss; AR amplification)	Chemotherapy (CDDP + DOX) + SBRT	Chemotherapy (CBDCA + ETP)	No	Died at 2 years and 10 months

Abbreviations: AR, androgen receptor, CBDCA, carboplatin; CDDP, cisplatin; CPT‐11, irinotecan; DOX, doxorubicin; DTX, docetaxel; ETP, etoposide; LOH, loss of heterozygosity; NA, not assessed or not applicable; PD, progressive disease; PSA, prostate‐specific antigen; SBRT, stereotactic body radiotherapy.

NEPC occurs in approximately 17% of metastatic CRPC and is increasingly recognized as t‐NEPC arising during prolonged androgen‐receptor–targeted therapy [6]. Loss or mutation of PTEN, TP53, and RB1 is implicated in neuroendocrine transdifferentiation [5, 6]. Clinically, NEPC shows relatively low PSA, elevated NSE, and resistance to androgen‐receptor–targeted therapy [6], all consistent with our case. Median cancer‐specific survival is approximately 7 months [7], and platinum‐based chemotherapy is most frequently used despite the absence of a standard [7, 8]. Analogous to small cell lung cancer, neuroendocrine tumors may be radiosensitive, and radiotherapy may have additive efficacy with chemotherapy [8]. Kasai et al. reported successful local control of urethral CRPC metastasis with CyberKnife radiosurgery [9].

In our patient, initial platinum‐based chemoradiotherapy reduced FDG uptake in the prostate, urethra, and ischium without subsequent recurrence; new disease at L5 was controlled by SBRT. Survival of 2 years 10 months substantially exceeded the reported median for NEPC. Although causality cannot be established from a single case, these findings suggest that combining platinum‐based chemotherapy with radiotherapy may have contributed to durable local control and prolonged survival, and that focal radiotherapy may add meaningful local control beyond chemotherapy alone. There are several limitations to this case report. First, although platinum plus etoposide is commonly used for NEPC by extrapolation from small cell lung cancer regimens, we selected cisplatin plus doxorubicin. This choice reflects institution‐specific practice and the treating physicians' preference in the context of multiple prior therapies for his concomitant metastatic renal cell carcinoma, including interferon‐alpha, interleukin‐2, and sorafenib, and does not represent a guideline‐based standard regimen. Second, at the terminal stage, new pulmonary and hilar lymph node lesions developed, but their origin could not be determined because histological confirmation was not feasible. Finally, although the urethral lesion was close to the prostate suggesting the possibility of direct invasion of t‐NEPC, the lack of continuity with the primary tumor on urethroscopy and imaging supported urethral metastasis rather than direct invasion.

In conclusion, platinum‐based chemoradiotherapy achieved durable urethral local control in t‐NEPC and warrants consideration in selected patients with NEPC‐associated urethral metastasis.

## Ethics Statement

The use of cisplatin plus doxorubicin, carboplatin, and etoposide for prostate cancer in this patient was reviewed and approved as an off‐label regimen (protocol number 622001) by the institutional Chemotherapy Committee of Jichi Medical University Saitama Medical Center prior to treatment.

## Consent

Written informed consent was obtained from the patient for publication of this case report and accompanying images.

## Conflicts of Interest

The authors declare no conflicts of interest.

## Data Availability

The data that support the findings of this study are available from the corresponding author upon reasonable request.

## References

[1] S. Ananthapadmanabhan , Z. Williams , H. Wang , A. Combes , V. Wong , and I. Thangasamy , “Prostate Cancer Recurrence in the Urethra With Low PSA,” Urology Case Reports 55 (2024): 102787. 39071853 10.1016/j.eucr.2024.102787 PMC11279688

[2] T. Masuda , T. Kosaka , K. Nakamura , et al., “Multiple Metastases of Androgen Indifferent Prostate Cancer in the Urinary Tract: Two Case Reports and a Literature Review,” BMC Medical Genomics 15, no. 1 (2022): 118. 35598018 10.1186/s12920-022-01267-z PMC9124419

[3] K. Sabharwal , A. Kumar , and A. Abrari , “Urethral Metastasis With Neuroendocrine Differentiation in a Patient With Prostate Cancer Treated With Hormone Deprivation,” Indian Journal of Urology 35, no. 1 (2019): 75–77. 30692729 10.4103/iju.IJU_175_18 PMC6334586

[4] T. Jindal , P. Pawar , and N. Subedi , “Unusual Presentation of Castrate‐Resistant Prostate Cancer With Urethral and Inguinal Nodal Metastasis,” Indian Journal of Urology 37, no. 1 (2021): 95–96. 33850366 10.4103/iju.IJU_285_20 PMC8033227

[5] L. Merkens , V. Sailer , D. Lessel , et al., “Aggressive Variants of Prostate Cancer: Underlying Mechanisms of Neuroendocrine Transdifferentiation,” Journal of Experimental & Clinical Cancer Research 41, no. 1 (2022): 46. 35109899 10.1186/s13046-022-02255-y PMC8808994

[6] N. Spetsieris , M. Boukovala , G. Patsakis , I. Alafis , and E. Efstathiou , “Neuroendocrine and Aggressive‐Variant Prostate Cancer,” Cancers 12, no. 12 (2020): 3792. 33339136 10.3390/cancers12123792 PMC7765615

[7] Y. Yamada and H. Beltran , “Clinical and Biological Features of Neuroendocrine Prostate Cancer,” Current Oncology Reports 23, no. 2 (2021): 15. 33433737 10.1007/s11912-020-01003-9 PMC7990389

[8] K. Suzuki , T. Terakawa , N. Jimbo , R. Inaba , Y. Nakano , and M. Fujisawa , “Clinical Features of Treatment‐Related Neuroendocrine Prostate Cancer: A Case Series,” Anticancer Research 40, no. 6 (2020): 3519–3526. 32487653 10.21873/anticanres.14340

[9] Y. Kasai , N. Sawada , T. Yamagishi , M. Kamiyama , T. Mitsui , and M. Takeda , “A Case of Urethral Metastasis of Castration‐Resistant Prostate Cancer Successfully Cured With CyberKnife Radiosurgery,” Urology Case Reports 33 (2020): 101346. 33102047 10.1016/j.eucr.2020.101346 PMC7573952

